# Thermosensitive Intravitreal In Situ Implant of Cefuroxime Based on Poloxamer 407 and Hyaluronic Acid

**DOI:** 10.3390/gels9090693

**Published:** 2023-08-28

**Authors:** Elena O. Bakhrushina, Anastasia I. Dubova, Maria S. Nikonenko, Viktoriya V. Grikh, Marina M. Shumkova, Tatyana V. Korochkina, Ivan I. Krasnyuk, Ivan I. Krasnyuk

**Affiliations:** 1 Department of Pharmaceutical Technology, A.P. Nelyubin Institute of Pharmacy, I.M. Sechenov First Moscow State Medical University (Sechenov University), Moscow 119048, Russia; bakhrushina_e_o@staff.sechenov.ru (E.O.B.); korochkina_t_v@staff.sechenov.ru (T.V.K.); krasnyuk_i_i@staff.sechenov.ru (I.I.K.); 2 Student of Educational Department, A.P. Nelyubin Institute of Pharmacy, I.M. Sechenov First Moscow State Medical University (Sechenov University), Moscow 119048, Russia; dubova_a_i@student.sechenov.ru (A.I.D.); nikonenko_m_s@student.sechenov.ru (M.S.N.); 3 Department of Analytical, Physical and Colloidal Chemistry, A.P. Nelyubin Institute of Pharmacy, I.M. Sechenov First Moscow State Medical University (Sechenov University), Moscow 119048, Russia; grikh_v_v_1@staff.sechenov.ru (V.V.G.); krasnyuk_i_i_1@staff.sechenov.ru (I.I.K.J.); 4 PHARMA-PREMIUM Scientific Educational Center, A.P. Nelyubin Institute of Pharmacy, I.M. Sechenov First Moscow State Medical University (Sechenov University), Moscow 119048, Russia

**Keywords:** in situ implant, intravitreal injection, cefuroxime, endophthalmitis, poloxamers, solid dispersion

## Abstract

The main method of treatment and prevention of endophthalmitis is a combination of intravitreal and topical administration of antibiotics, such as cefuroxime moxifloxacin or vancomycin. However, this method is ineffective due to the rapid elimination of the drug. This problem can be solved with the help of intravitreal in situ injection systems, which are injected with a syringe into the vitreous body and provide prolonged action of the drug at the focus of inflammation. Under the influence of temperature, the liquid drug undergoes a phase transition and turns into a gel after injection. This ensures its prolonged action. The study aimed to develop an intravitreal in situ cefuroxime delivery system for the treatment of endophthalmitis based on a thermosensitive biodegradable composition of poloxamer 407 and hyaluronic acid. A combination of poloxamer Kolliphor^®^ P407, Kolliphor^®^ P188, and PrincipHYAL^®^ hyaluronic acids of different molecular weights was used as a delivery system. The potency of cefuroxime solid dispersion with polyvinylpyrrolidone-10000, polyethylene glycol-400, and polyethylene glycol-1500 in a 1:2 ratio was studied for prolonged action compared to cefuroxime substance. The experimental formulations were studied for the parameters of gelation temperature in a long-term test (4 months), pH, and release of cefuroxime using dialysis bags. To study the distribution parameter in the vitreous body, an in vitro model (1/13) was developed, which was a hollow agar sphere filled with 1% (*w*/*v*) polyacrylate gel. For the superior formulations, a HET-CAM test (chorioallantoic membrane test) was performed to determine the absence of irritant effects. According to the study results, a formulation containing a solid dispersion of cefuroxime:PEG-400 (1:2), the matrix of which contained 18% (*w*/*v*) Kolliphor^®^ P407 poloxamer, 3% (*w*/*v*) Kolliphor^®^ P188 poloxamer, and 0.5% (*w*/*v*) hyaluronic acid (1400–1800), was selected. This sample had an average gelation temperature of 34.6 °C, pH 6.7 ± 0.5, and a pronounced prolonged effect. Only 7.6% was released in 3 h of the experiment, whereas about 38% of cefuroxime was released in 72 h. No irritant effect on the chorioallantoic membrane was observed for any formulations studied.

## 1. Introduction

Endophthalmitis is a severe form of eye inflammation caused by intraocular infection that can lead to irreversible loss of vision. In most cases, this disease is caused by a penetrating eye injury or ophthalmic surgery [1,2].

In terms of the etiology of the disease, bacterial and fungal endophthalmitis are distinguished [3,4,5]. Bacterial endophthalmitis, caused by Staphylococcus epidermidis, Staphylococcus aureus, Streptococcus, Enterococcus, and Gram-negative bacilli, is the most common [6,7,8].

According to meta-analyses published between 2017 and 2022, the efficacy of intracameral and intravitreal injections with antibiotics such as cefuroxime, moxifloxacin, and vancomycin is comparable to that of pars plana vitrectomy in the treatment of postoperative (post-cataract) endophthalmitis [9,10,11]. Meanwhile, the introduction of cefuroxime and moxifloxacin has shown minimal toxic effects, whereas vancomycin in some cases had a toxic effect on the retina [12]. Currently, the use of cefuroxime, a second-generation cephalosporin that is active against a broad spectrum of Gram-positive and Gram-negative microorganisms, is still the standard of prophylaxis against endophthalmitis [13].

Although topical application of eye solutions remains the easiest and most common way to administer ocular drugs, this method is ineffective due to its low bioavailability, which is approximately 5% [14]. The presence of protective physiological mechanisms such as lacrimation, nasolacrimal drainage, and blinking limits the penetration of drugs into the ocular tissues [15]. Ophthalmic gels and ointments are used to increase the retention time of drugs on the ocular surface. However, the disadvantages of this method are decreased dosing accuracy, blurred vision, crusting of the eyelids, and increased tear production [16].

Intravitreal administration today remains one of the main methods used by ophthalmologists to deliver drugs to the focus of inflammation [17]. However, over the past two decades, considerable experience has been accumulated demonstrating a significant increase in the risk of endophthalmitis when intravitreal injections are performed under inappropriate conditions or protocol violation [18]. Reducing the frequency of intravitreal injections during therapy by creating prolonged delivery systems designed to release the active ingredient for more than 48 h can be an effective measure to reduce the risk of secondary endophthalmitis after medical manipulation [19].

In situ systems are modern alternatives to conventional drugs, combining important advantages such as the convenience of administration, stimuli-responsive phase transition, and prolonged action [20,21]. Thermo- and photosensitive compositions are the most widely used for intravitreal injection of in situ systems [22,23]. Unlike photosensitive implants, thermosensitive in situ systems do not require any additional medical manipulations to ensure phase transition after injection. However, the selection of an optimal thermosensitive polymer (or polymer compositions) that satisfies the experimentally established parameters is still a challenge. Many researchers have used methods to synthesize thermosensitive polymers to achieve the desired phase transition temperature, biodegradation parameters, etc., achieving impressive results [23,24]. However, directed synthesis unfortunately only aggravates the problem of in situ systems. It is associated with the complexity of polymer standardization, as well as the reproducibility of methods and technology transfer, which does not contribute to achieving the product on the market.

Many researchers are focusing on a well-known thermosensitive component, the block copolymer, consisting of polypropylene glycol and polyethylene glycol (PEG), poloxamer 407, which is a commercially available and standardized excipient that is also approved for parenteral use by the FDA [25,26]. Thus, a study by Yu Yu et al. (2015) proved the complete biocompatibility of a thermosensitive in situ system based on thiolated dextran and hyaluronic acid derivative [27]. Awwad S. et al. (2019) reported the experience of successful upgrading of thermosensitive but non-biodegradable N-isopropylacrylamide with acrylated hyaluronic acid as a biodegradable macromolecular crosslinking agent [28].

The use of other gelling agents in in situ compositions helps to adjust the temperature of the phase transition. Moreover, the addition of high-molecular compounds to poloxamer P407 allows its concentration to be reduced without changing its gel-forming features. Thus, the use of hyaluronic acid is preferable not only because of the desired gelation temperature but also thanks to biological affinity for the site of administration [29,30]. The development of a hyaluronic acid-based intravitreal in situ implant was carried out by Yu Yu et al. [27]. The results demonstrated the in situ biocompatibility of the system, as well as its ability to maintain the release of the active ingredient in the vitreous body at therapeutically relevant concentrations for at least 6 months.

Thus, the study aimed to develop an intravitreal in situ cefuroxime delivery system for the treatment of endophthalmitis based on a thermosensitive biodegradable composition of poloxamer 407 and hyaluronic acid.

## 2. Results and Discussion

In the course of a long-term study, the temperature and time of the phase transition of polymer matrices based on Kolliphor^®^ P407 poloxamer, Kolliphor^®^P188, high-molecular hyaluronic acid, and polyethylene glycol 1500 were studied. The compositions are presented in Table 1.

The phase transition temperature was measured weekly and stopped when the gelation temperature reached a value close to room temperature, which was assumed to be 25 °C. The initial values of phase transition temperatures for freshly prepared compositions were determined to be in the range of 30 to 42 °C. However, while the samples were stored at temperatures between 5 and 8 °C, the gelation temperature gradually decreased and reached a plateau in the range of 32–38 °C. According to published data, such a range of temperatures can be considered sufficient for intravitreal implantation, as such values are within a safe range from storage and room temperature and do not exceed the physiological temperature in the vitreous body [13,31]. Thus, the aim was to screen the formulations for the longest-maintained phase transition temperature within an acceptable range for the developed delivery system (Table 2). Compound **8** ceased to satisfy the experimental design conditions at the 18th week of storage. Compound **3**, which did not initially have a temperature close to the physiological site of administration, ceased to satisfy at the 19th week of storage. Compounds **4**, **5**, **2**, and **1** deviated from the requirements of the experimental design conditions in the 20th, 21st, and 22nd week, respectively. Compositions 6 and 7, which contained 18% (*w*/*v*) Kolliphor^®^ P407 (P407) poloxamer and 2.5% (*w*/*v*) and 3.0% (*w*/*v*) Kolliphor^®^ P188 (P188) poloxamer, respectively, and 0.5% (*w*/*v*) high molecular weight hyaluronic acid (HA), were stable the longest. Compound **7** maintained the temperature within the required range from week 9 to week 40 (31 weeks). Compound **6** maintained temperature from the 8th to the 38th week of measurements (30 weeks).

Thus, based on preliminary studies, composition 6 was chosen because it supported phase transition within the permissible experimental design range for the longest period and also had a lower SD compared to compound **7**’s SD (Table 3). Further, compositions C1, C2, and C3, containing hyaluronic acids of various molecular weights, along with the active component in the active concentration (1.0%, *w*/*v*) and ascorbic acid as an antioxidant, were developed. The characteristics of compositions C1, C2, and C3 are shown in Table 3.

The transformation of the prepared samples into a gel occurred on average at a temperature of 36.4 °C in 3 min, which allowed for a phase transition at a physiological temperature of the eye cavity. It should be noted that after the addition of cefuroxime, the time of the phase transition was extended by an average of three times, regardless of the hyaluronic acid used.

The pH values of the formulations studied met the requirements for ophthalmic forms and ranged from 6.62 to 6.89. Transparency, which is measured by light transmittance in the range of 200 to 800 nm, was greater than 88%.

The in vitro model created during the experiment was used to assess the degree of diffusion of the studied compositions in the eye cavity. To date, there are several known in vitro models of the vitreous body proposed by scientists to evaluate the performance of intravitreal implants such as distribution, diffusion, and release of the active ingredient [32,33,34,35,36,37]. Often in vitro, models are 3D-printed spheres filled with polyacrylate and agar with hyaluronic acid added. In terms of viscosity and rheological characteristics, they correspond to the vitreous body of the human eye. Some of the models provide for physiological-like rotation. The use of dissolution testers for analysis (standardized USP apparatuses 4 and 7) described in several publications does not meet the requirement of biorelevance.

In the proposed model, the density of polyacrylate gel corresponded to the characteristics of the human vitreous body and was 0.9956 g/cm3. The pH of the resulting gel was 7.4, which corresponded to the pH values of the vitreous body [13]. The agar shell kept the shape of the model. It also ensured the convenience of inserting the samples inside the model.

**Figure 1:**
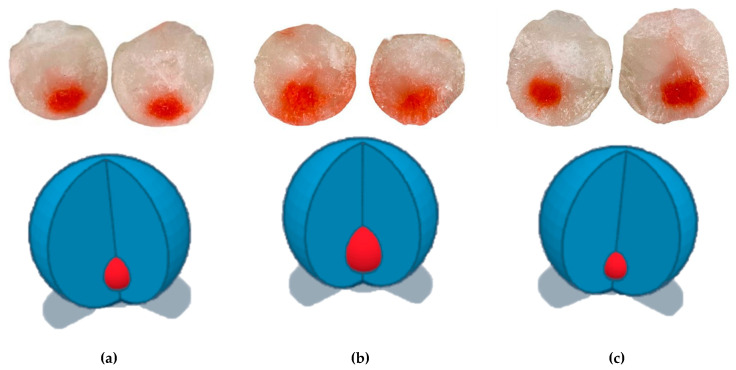
Distribution of injected samples (marked in red) C1—containing hyaluronic acid mixture (**a**), C2—low molecular weight (**b**), or C3—high molecular weight (**c**) hyaluronic acids in an in vitro model.

After measuring the volume of colored sample distribution, building a 3D model, and visual assessment (Figure 1), it was concluded that the composition with the addition of low-molecular hyaluronic acid demonstrated the largest volume of diffusion (25.3% of the model volume). Samples containing high-molecular hyaluronic acid and a mixture of hyaluronic acids showed a much smaller distribution (7.0% and 8.6% of the model volume).

The findings are consistent with the results of Thakur SS. et al. (2020), who created on in vitro model of the vitreous body for sodium fluorescein [38]. In addition, a study by Kim HM et al. (2020) on rabbit eyes in vivo demonstrated a similar relationship between the distribution of particles of different sizes in the vitreous body. At the same time, the authors also noted the effect of the size of the injected particles on retention and excretion from the vitreous body. Thus, additional in vivo studies will be required to provide a complete and reliable understanding of the elimination kinetics of the drug under development at the site of administration [39].

After assessing the results of measuring the temperature of the phase transition of the samples, it was concluded that the composition with the addition of high-molecular hyaluronic acid (C3) was the most stable. The standard deviation of the gelation temperature sample was 4.14. In compositions with the addition of low-molecular hyaluronic acid (C2) or a mixture of hyaluronic acids (C1), this figure was 4.38 and 6.13, respectively.

**Figure 2:**
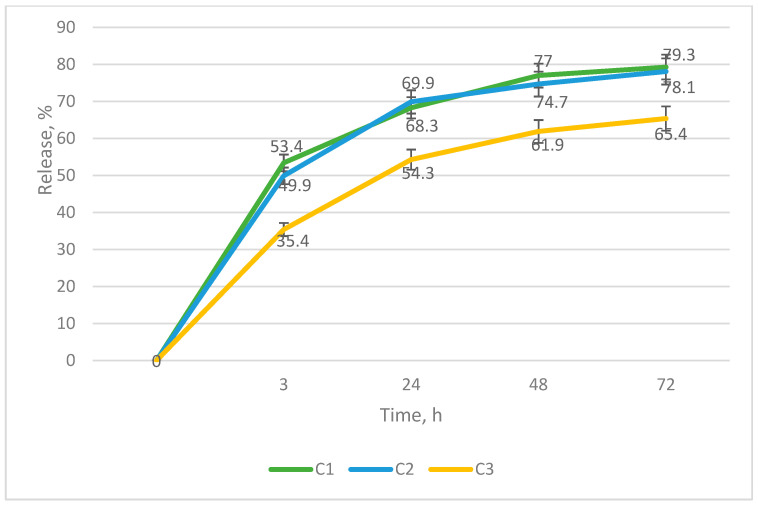
Graph of cefuroxime release from polymer matrix (n = 5).

The sample containing high-molecular hyaluronic acid (C3) demonstrated the lowest release. The percentage of release in the third hour of dialysis was 35.4 (SD = 1.28), 54.3 (SD = 0.75) after 24 h, 61.9 (SD = 1.36) after 48 h, and 65.4 (SD = 0.99) after 72 h (Figure 2).

At the next step of the study, the possibility of incorporating solid dispersion of cefuroxime into the developed delivery systems for intravitreal implantation was studied. The values of the phase transition temperature, pH, and distribution volume of the obtained compositions were similar to those obtained earlier for samples containing free cefuroxime (Table 4). It should also be noted that the standard deviation of the temperature values for these samples during the long-term study was smaller than for the C1–C3 compositions containing free cefuroxime.

**Figure 3:**
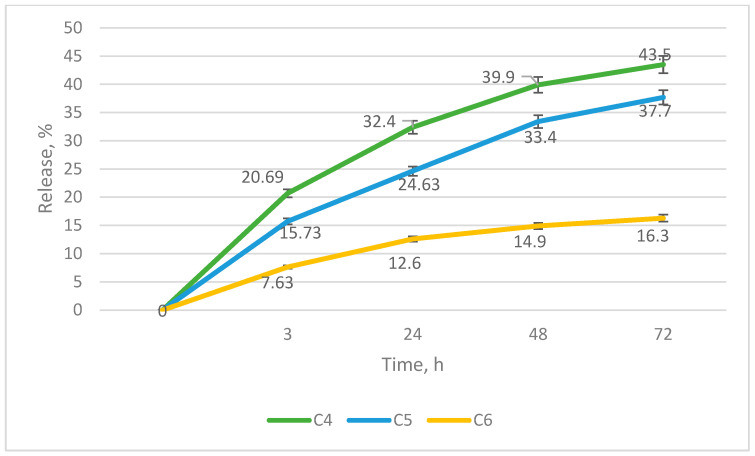
Graph of cefuroxime release from solid dispersion polymer matrix (n = 5).

As a result of studying the release of the active substance, the data presented in the graph (Figure 3) were obtained. According to the results of the preliminary evaluation of technological characteristics and release, sample C5 was selected for further study. The percentage of release in the third hour of dialysis was 15.7 (SD = 0.98), after 24 h was 24.6 (SD = 1.02), after 48 h was 33.7 (SD = 1.73), and after 72 h was 37.7 (SD = 0.90) (Figure 3).

Thus, the feasibility of incorporating a solid dispersion of cefuroxime into a thermosensitive system was demonstrated in the experiments performed. Compositions C4–C6 showed a significant modification of the release, as well as greater stability of the phase transition temperature measured in long-term tests. It is worth mentioning that the incorporation of solid dispersion did not significantly affect the values of gelation temperatures and did not change the pH of the formulations.

To date, according to the database of medical publications PubMed, there are very limited works known in the world related to the incorporation of solid dispersions of thermosensitive in situ systems based on poloxamers [40]. One of the most recently published studies is by Zhang C. et al., where solid dispersion of disulfiram as part of an in situ poloxamer-based system is used for cataract treatment [41]. In a comparative dissolution test, it was shown that the release of disulfiram from the solid dispersion placed in the poloxamer matrix was almost twice the release of free disulfiram from the in situ system in the first hour. The authors of the study suggest that disulfiram in the solid dispersion was in a highly dispersed state, which significantly improved its solubility in water compared to crystalline disulfiram, which is practically insoluble in water.

For the examined solid dispersion of cefuroxime, the inverse correlation was observed. The release of the active substance during the first three hours of the test decreased on average by 1.5–2 times compared to free cefuroxime, which is a significantly prolonged effect. This is probably because the solid dispersion of cefuroxime contains a complex of water-soluble compounds with high molecular weights in their composition [42]. During the release test, at first, the solvent against the concentration gradient tended to enter the polymer dispersion matrix through the dialysis membrane, which promoted the breakage of hydrogen bonds of the complex, allowed the release of the antibiotic from the matrix, and permitted its diffusion into the receptor medium. Free cefuroxime, being amorphous and very soluble in water, diffused much faster into the solvent compared to cefuroxime in the polymer matrix.

**Figure 4:**
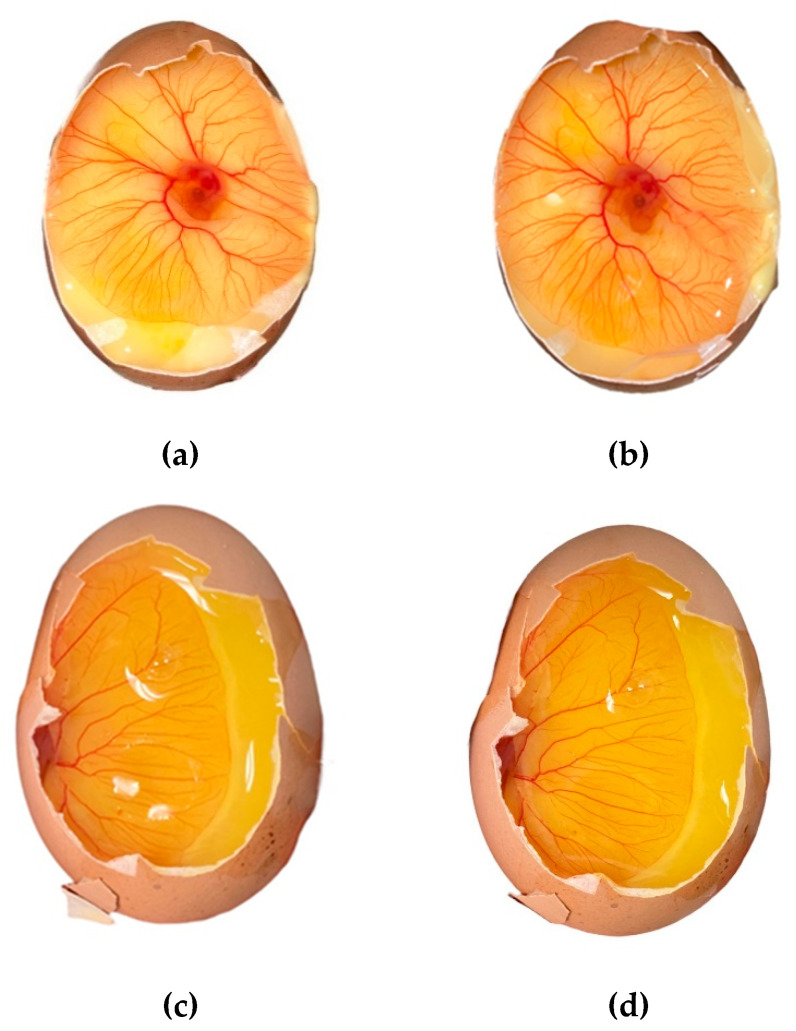
Chorioallantoic membrane before and after application of the studied samples C3 and C5; (**a**) control for composition C3, (**b**) after application of composition C3, (**c**) control for composition C5, (**d**) after application of composition C5.

The following results were obtained after the test on the chorioallantoic membrane: After applying the samples to the membrane surface, no change in its color was observed, and there was no hemorrhage (Figure 4). Thus, the absence of the irritating effect of the studied compositions containing both free cefuroxime and solid dispersion of cefuroxime was proven.

## 3. Conclusions

In the study, the compositions of experimental samples were justified. As a result of the screening of samples by phase transition temperature, the most stable composition containing 18% (*w*/*v*) poloxamer Kolliphor^®^ P407, 3% (*w*/*v*) poloxamer Kolliphor^®^ P188, and 0.5% (*w*/*v*) high-molecular-weight hyaluronic acid was selected. The incorporation of free cefuroxime did not allow the desired parameters of prolongation of the effect during release to be achieved. For composition C3, characterized by the most prolonged release, the release of cefuroxime into the dialysis medium in 3 h was about 36%. The incorporation of cefuroxime into the composition of solid dispersion was proposed, which had a positive effect on the release kinetics evaluated in vitro. Thus, in 3 h of release from the formulation containing 7.6%, the release reached 38% of cefuroxime in 72 h. The chorioallantoic membrane test confirmed the absence of irritating effects on the mucosa for all formulations developed, which provides a reason to pursue further study of this formulation in long-term in vitro as well as in vivo trials.

## 4. Materials and Methods

### 4.1. Materials

The following materials were used in this study: poloxamer Kolliphor P407 (BASF SE, Ludwigshafen, Germany); poloxamer Kolliphor P188 (BASF SE, Ludwigshafen, Germany); hyaluronic acids PrincipHYAL (1400–1800), PrincipHYAL (400–600), and PrincipHYAL (Cube3) (ROELMI.HPC, Origgio, Italy); PEG 1500 (BASF SE, Ludwigshafen, Germany); PEG 400 (BASF SE, Ludwigshafen, Germany); polyvinylpyrrolidone-10000 (PVP) (BASF SE, Ludwigshafen, Germany); ascorbic acid (Acros Organics, Geel, Belgium); cefuroxime (Kraspharma, Krasnoyarsk, Russia); food agar (Dr. Oetker, Belgorod, Russia); sodium polyacrylate (RusHim, Moscow, Russia); and food coloring (Kreda, Izhevsk, Russia).

### 4.2. Methods

Kolliphor P407 and Kolliphor P188, hyaluronic acids, and polyethylene glycol at different concentrations were dissolved in purified water when stirred on a digital IKA C-MAG HS 7 magnetic stirrer (Germany) to obtain polymer matrices. Stirring was continued until homogeneous compositions were formed.

In the first stage, samples based on high-molecular hyaluronic acids were screened throughout the year by gelation temperature for the purpose of selecting the most optimal concentrations of Kolliphor P188 and PEG. The composition with the most stable gelation temperature was chosen for further research. The time range of measurements was chosen based on our own published studies, which showed the necessity of long-term (12 to 52 weeks) studies to conclude the degree of stability of the gelation temperature index of samples based on poloxamer 407 and additional polymers [43,44].

After the production of similar compositions, cefuroxime was introduced as an active substance at a concentration of 10 mg/mL (*w*/*v*) [13]. For this purpose, 0.3 g of cefuroxime was dissolved in 30 mL of purified water under magnetic stirring. After complete dissolution of the active component, Kolliphor^®^ P407 poloxamer (P407), Kolliphor^®^ P188 poloxamer (P188), hyaluronic acid, and ascorbic acid were added to the composition. Three types of hyaluronic acids were used for the manufacture of samples: low-molecular (C2), high-molecular (C3), and a combination (C1).

The phase transition temperature of the obtained compositions was studied by using the ultrasonic bath ODA-MH13 (Shen Zhen Derui Ultrasonic Equipment Co., LTD, Shenzhen, China) heated up to 50 °C, a temperature probe, and a stopwatch for 4 months with a frequency of once a week, according to the method described by Salem H.F. et al. (2019) [45]. Measurements for each compound were performed five times consecutively. The pH values of those compositions were determined by using the stationary pH meter Starter 2100 (OHAUS, Parsippany, NJ, USA).

All of the obtained compositions at storage temperature (5 °C) and room temperature were transparent colorless liquids without mechanical inclusions by visual evaluation on a black-and-white background. Additionally, the transparency test of the compositions was carried out by a technique involving spectrophotometric measurement of the transmittance value in the wavelength range of 200 to 800 nm [46]. The measurements were performed on an Agilent Cary 60 spectrophotometer (Agilent Technologies Inc., Santa Clara, CA, USA) and compared with purified water [46].

The release of cefuroxime from experimental compositions was studied by using a dialysis membrane with 200 mL of lacrimal fluid as an environment, which was made by dissolving NaCl (0.67%, *w*/*v*), NaHCO3 (0.20%, *w*/*v*), and CaCl2 (0.008%, *w*/*v*) in distilled water [47]. This method has been widely used in many experiments to evaluate the performance of designed intravitreal implants [48]. The receptor medium volume was chosen by taking into account the volume of liquid passing through the vitreous body per day [49]. For the test, 0.5 mL of the drug was placed on an OrDial D14b dialysis membrane with a pore size of 12–14 kDa (OrangeScientific, Braine-l’Alleud, Belgium). The membrane was fixed on the end of a dialysis tube. The other end was capped using laboratory paraffin film. The dialysis cell was fixed on a glass vessel with the receptor medium and placed in a climate chamber maintaining a temperature of 37 ± 0.5 °C. Sampling was performed at a frequency that was set by the design of the experiment. The sample volume was 5 mL. The sampled volume was replaced by the receptor medium. The test was repeated five times, and the release profiles were plotted against the averaged results.

Quantitative determination of cefuroxime in the release environment was determined by using the spectrophotometry method at a wavelength of 278 nm according to literature data corresponding to the characteristic peak of cefuroxime. The technique was validated by specificity, linearity, and precision parameters using two spectrophotometers: UNICO 2100 (UNICO, Union City, NJ, USA) and Agilent Cary 60 (Agilent Technologies Inc., Santa Clara, CA, USA). Statistical processing of the results of all studies was carried out using IBM SPSS Statistics 24.0.

To prolong the release of the active substance, compositions containing solid dispersion of cefuroxime were manufactured, for the manufacture of which the calculated amount of cefuroxime and polymer (1:2) was dissolved in purified water (15 mL) until the substances were completely dissolved and a homogeneous transparent solution was formed. Then, the resulting solution was evenly applied to a metal tablet and the solvent was evaporated at a constant airflow at a temperature of 60 ± 5 °C for 2 h until a homogeneous film of solid dispersion cefuroxime:polymer was obtained. Polyvinylpyrrolidone-10000 (PVP) (C4), polyethylene glycol-400 (PEG-400) (C5), and polyethylene glycol-1500 (PEG-1500) (C6) were used as polymers.

### 4.3. Development of In Vitro Model

To study the distribution of the studied compositions during intravitreal administration, an in vitro model was developed, which was an agar sphere filled with polyacrylate gel imitating a vitreous body [50].

A sample of 9% (*w*/*v*) agar (300 mL) was prepared to obtain the model with the addition of 0.9% (*w*/*v*) low-molecular-weight hyaluronic acid PrincipHYAL (400–600). The solution was prepared at 80 °C, stirring constantly on IKA^®^ C-MAG HS 7 digital magnetic stirrer (Germany). The resulting mixture was poured into spherical silicone molds (50 mL). After solidification, the agar spheres were removed from the form and the internal contents were removed from them.

For the preparation of 1% (*w*/*v*) polyacrylate gel, sodium polyacrylate was dissolved in a buffer solution while stirring on a IKA^®^ C-MAG HS 7 digital magnetic stirrer (Germany). The density of the gel was set by the mass–volume method. Its pH was also determined using the Starter 2100 pH meter (OHAUS, Parsippany, NJ, USA).

After heating the polyacrylate gel on ultrasonic ODA-MH13 (Shen Zhen Derui Ultrasonic Equipment Co., LTD, Shenzhen, China) to physiological temperature, the gel was poured inside the agar shell. Without waiting for it to cool down, 0.5 mL of the studied samples, pre-painted, was introduced into the model with a syringe. The model was placed in a climate chamber (maintained temperature of 37 ± 1 °C) for 30 min, after which it was frozen at −20 °C for 24 h. After one day, the model was removed from the freezer and cut into 4 equal parts. The volume of distribution of compositions along the cavity of the model was measured. The results of the experiment were photographed.

The distribution of composition along the model was observed visually after building a 3D model in Tinkercad (Autodesk, Inc., San Francisco, CA, USA).

### 4.4. IV. Chorioallantoic Membrane Test

To check for irritation of the mucous membrane, a HET-CAM test (chorioallantoic membrane test) was carried out [51]. The essence of the method is as follows: Embryonated chicken eggs weighing 50.0–60.0 g without defects are incubated at a temperature of 37 ± 0.5 °C for 3 days and periodically turned over. After that, in the equatorial position on the shell, the hole is made in such a way that the chorioallantoic membrane is visible, on which the studied samples are placed and evaluated according to the parameters presented in the table (Table 5).

## Figures and Tables

**Table 1:** Compositions of experimental samples (*w*/*v*).

Ingredient	1	2	3	4	5	6	7	8
Cefuroxime	-	-	-	-	-	-	-	-
P407	18%	18%	18%	18%	18%	18%	18%	18%
P188	0.5%	1%	-	2%	1.5	2.5%	3%	-
HA	0.5%	0.5%	0.5%	0.5%	0.5%	0.5%	0.5%	0.5%
PEG 1500	-	-	-	-	-	-	-	1.5%
Ascorbic acid	0.5	0.5	0.5	0.5	0.5	0.5	0.5	0.5
Purified water	ad 20 mL							

**Table 2:** Change in temperatures (°C) of the phase transition of experimental samples (n = 5).

Composition	Critical Points of Change in the Indicator (Weeks)								Average Temperature of the PHASE transition (°C)	SD
	1	18	19	20	21	22	39	41		
1	31.4	27	26	27.8	27.2	24 *	-	-	28.2	3.79
2	32.8	28.6	28.5	28.8	25.3 *	-	-	-	30.7	2.08
3	29.9	27.6	25 *	-	-	-	-	-	27.9	1.54
4	33.1	31.4	30.9	25 *	-	-	-	-	33.2	1.71
5	32.7	28	27	24.5 *	-	-	-	-	30.9	5.22
6	43.9	34	34	36	34	33	25 *	-	35.0	5.34
7	44.3	38	37	37	36	36	31	25 *	36.4	6.40
8	35	25 *	-	-	-	-	-	-	31.7	3.81

* Critically low temperature, after which measuring was stopped.

**Table 3:** Composition, average phase transition temperature, pH, and distribution volume of experimental samples C1–C3 (n = 10).

Composition	Type of Hyaluronic Acid	Average Temperature of the Phase Transition (°C)	SD	pH	Diffusion Volume (% of Model Volume)
**C1**	Hyaluronic acid (Cube3)	34.8	6.13	6.89	8.6 ± 1.65
**C2**	Hyaluronic acid (400–600)	37.4	4.38	6.65	25.3 ± 2.4
**C3**	Hyaluronic acid (1400–1800)	37.1	4.14	6.62	7.0 ± 1.9

**Table 4:** Composition, average phase transition temperature, pH, and volume of distribution of experimental samples C4–C6 (n = 10).

Composition	Type of Solid Dispersion	Average Temperature of the Phase Transition (°C)	SD	pH	Diffusion Volume (% of Model Volume)
**C4**	PVP (K15)	36.3	2.72	6.72	6.3 ±1.5
**C5**	PEG 400	34.6	2.69	6.69	8.0 ± 2.3
**C6**	PEG 1500	35.7	3.19	6.70	5.6 ± 2.0

**Table 5:** Parameters for assessing the results of the experiment on the chorioallantoic membrane.

Range	Parameter
0	The color of the membrane has not been changed, no hemorrhage
1	Noticeable disappearance of membrane color, no hemorrhage
2	Partial loss of membrane color, a hemorrhage
3	Complete color loss, hemorrhage over the entire surface of the film’s contact with the membrane

## Data Availability

The authors can provide any necessary information on the study upon request.
